# Use of the femoral vein ('groin injecting') by a sample of needle exchange clients in Bristol, UK

**DOI:** 10.1186/1477-7517-2-6

**Published:** 2005-04-15

**Authors:** John Maliphant, Jenny Scott

**Affiliations:** 1 Bristol Drugs Project, 11 Brunswick Square, Bristol, BS2 8PE, UK; 2 Dept Pharmacy & Pharmacology, University of Bath, Bath, BA2 7AY, UK

## Abstract

**Background:**

Use of the femoral vein for intravenous access by injecting drug users (IDUs) (commonly called 'groin injecting') is a practice that is often observed but on which little is written in the literature. The purpose of this study was to describe self-reported data from a sample of groin injectors on the natural history and rationale regarding their groin injecting, to inform future research and the development of appropriate harm reduction strategies.

**Methods:**

A convenience sample of groin injectors willing to participate in a semi-structured interview were recruited through the Bristol Drugs Project Harm Reduction Service. The interviews were conducted over the period of one week. Data on transition to groin injecting, rationale for use and incidence of problems were collected.

**Results:**

Forty seven IDUs currently injecting in their femoral vein ('groin') were interviewed, 66% (n = 31) male and 34% (n = 16) female. Their mean age was 31 yrs (range 17 to 50 yrs; SD = 7.7). The mean length of time since first injecting episode was 9.6 yrs (range 6 mths to 30 yrs; SD = 7.0). The mean length of time since use of the groin began was 2.6 years (range 1 mth to 15 yrs; SD = 3.3). The mean length of time between first injection and first use of the groin was 7.0 yrs (SD = 7.0). One person had used no other area for venous access prior to using the groin, nine people had used one, nine people had used two, 10 people had used three, five people had used four and 13 people had used more than four areas. The main reason given for starting to inject in the groin was that 'no other sites were left'. However further discussion identified this meant no other *convenient *sites were accessible. Practises such as the rotation of injecting sites, as advocated in many harm reduction leaflets, were reported to be difficult and unreliable. The risk of missing the vein and subsequently losing the 'hit' was considered high. Use of the non-dominant hand to administer injections was problematic and deterred rotation between arms. The groin site was reported to be convenient, provide quick access, with little mess and less pain than smaller more awkward veins. The formation of sinuses over time facilitated continued use of the groin. Approximately two thirds of participants had experienced difficulty gaining IV access at their groin. Common problem included scar tissue occlusion, swelling and pain. Some reported infections and past history of deep vein thrombosis.

**Conclusion:**

Use of the groin was perceived to be convenient by the study group. Problems following safer injecting advice were identified, including dexterity difficulties leading to fear of losing the 'hit'. Health problems at the groin site did not deter use. These results suggest further qualitative work is needed to explore the difficulties in following safer injecting advice in more detail and inform the development of more appropriate advice. Further quantitative work is necessary to establish the prevalence of groin injecting amongst IDUs and the incidence of associated problems. There is a need for a longitudinal study to examine the relationship between injecting technique and loss of patency of veins. If protective factors could be identified, evidence-based safer injecting advice could be established to preserve peripheral veins and reduce use of the groin site, which is high risk and associated with serious adverse consequences.

## Background

The physical health complications of injecting drug use are well documented in the literature (e.g.[1-6]). The injections used by injecting drug users (IDUs) are non-sterile and not subject to quality control. This, coupled with frequent, chronic venous administration is associated with damage to the vascular structure. Vascular damage commonly begins with thrombophlebitis, leading to vein sclerosis and loss of patency [1,3], rendering the vein unusable. This leads to the IDU seeking other useable points of intravenous (IV) access.

There is little research literature reporting patterns of vascular access in IDUs. If the natural history of IDU injecting patterns was better understood, effective strategies to protect vascular health could be explored. A paper by Darke et al [7] describes a pattern of use of various injecting sites over time, identified amongst a sample of injecting drug users in Sydney, Australia. The authors report most injectors began their injecting careers using the cubital fossa (inner crook of the arm), with a pattern of progression through forearm (after a median of two yrs from first injection), upper arm (3.5 yrs from first injection), hand (4 yrs from first injection), neck, feet, leg (all 6 yrs after first injection) and finally groin and peripheral digits (both 10 yrs from first injection). This suggests that use of the groin amongst this sample was reserved as a 'last resort' with other points of access being selected first.

The use of the femoral vein in the groin by IDUs is of concern. It is linked with increased risk of vascular complications such as deep vein thrombosis (DVT), leg ulcers and vascular insufficiency. Its close proximity to the femoral artery and nerve also poses the risk of inadvertent trauma to these sites. Arterial injection is associated with arterial spasm and arterial thrombus formation [8].

The literature contains reports of adverse consequences from use of the groin site but little qualitative study of the factors that motivate this practice. The primary purpose of this study was to inform service development at Bristol Drugs Project (BDP) and compare the findings with that of Darke et al^5^. However the findings of this work are of interest to the wider harm reduction community because little is written in the literature about groin injecting. This study begins to shed some light on factors that motivate this practice. It also suggests future areas for research around groin injecting in order to inform the development of evidence-based safer injecting advice.

## Methods

### Location

This study was undertaken in Bristol, which is the largest city (pop. 382,000) in the South West region of England, UK. Participants were clients of BDP, which is a voluntary sector drug service. BDP is the only needle exchange and harm reduction agency in the city, but there are also pharmacy-based exchanges.

### Recruitment

A convenience sampling method was used. Willing participants were recruited from attendees at BDP needle exchange base which is a fixed site service, and the Mobile Harm Reduction Service, which is a vehicle providing outreach needle exchange services across the city. Data was collected over a period of one week in 2004 by the same interviewer in all cases. All clients who used the needle exchange services staffed by the interviewer were invited to take part in the study. Participants were guaranteed strict confidentiality and data was collected anonymously.

### Data collection

Data was gathered using a short semi structured interview based on a series of questions derived from previous discussions amongst needle exchange clients and staff. It explored injecting history and whether the person had ever or was currently experiencing problems using their groin. The study was reviewed and approved by the BDP management board. The interview was conducted after the needle exchange transaction was completed. Verbal consent was obtained. Data was recorded on a tick-box data collection form by the interviewer and by additional note writing.

### Analysis

Data was coded and input into SPSS for Windows (v. 12, SPSS inc. Illenois, 2003) for analysis where appropriate. Descriptive data was analysed to identify emergent themes.

## Results

### Incidence of use of the groin and demographics

The interview took approximately 10 minutes. A total of 92 clients were interviewed as part of the wider review and 47 (51%) of these were currently injecting in their groin. None of those who were not injecting in the groin presently had ever done so in the past. Of those injecting in their groin, 66% (n = 31) were male and 34% (n = 16) were female. The mean age of the groin injectors was 31 yrs (SD = 7.7), with the youngest being 17 and the oldest being 50 yrs. Twelve (26%) of the groin injectors were between 17–24 yrs, 29 (62%) were between 25 and 39 yrs and 6 (13%) were between 40–50 yrs.

### Length of time injecting

The mean length of time since first injecting episode was 9.6 yrs (SD = 7.0), with the shortest time since first injecting episode being 6 months and the longest time being 30 yrs. Seventeen (36%) had been injecting 5 yrs or less, 12 (26%) had been injecting between 6 and 10 yrs, 10 (21%) had been injecting 11–15 yrs, 6 (13%) had been injecting 16–20 yrs, one (2%) had been injecting for 25 years and one (2%) for 30 years.

### Length of time of groin injecting

The mean length of time since use of the groin site began was 2.6 years (SD = 3.3), with the shortest time being 0.08 years (1 month) and the longest time being 15 years. The mode length of time was 2 years, reported by 6 people (13%).

### Time from first injecting episode to first use of the groin

The mean length of time between first injection and first use of the groin for IV access was 7.0 yrs (SD = 7.0). Two people (4%) had been using the groin for IV access since they first began injecting, both were male. One had tried to inject into the arms unsuccessfully prior to using the groin, so switched to the groin straight away. This person was very thin with no visible veins, so chose to use the groin to ensure IV access. The other person had not tried any other injecting sites prior to the groin, as all his associates who were injecting at the time were using the groin, encouraging him not to attempt to try any others first. Twenty five people (53%) had begun using the groin within 5 years from their first injection and 11 (23%) had begun using the groin 5 or more yrs but less then 10 years since their first injection. Eleven people (23%) had begun using the groin 10 or more yrs since their first injection. The longest time between first injection and use of the groin was 23 yrs in a male who had begun injecting 25 yrs ago but only started using the groin 2 yrs ago.

### Areas used prior to the groin

People were asked to report which areas they had injected into prior to using the groin. One (2%) person had used no other area for venous access prior to using the groin and had been using this site for 15 years. Nine (19%) people had used one other area and in all cases this was the cubital fossa (inner crook of the arm). Nine (19%) people had used two areas prior to the groin, all of these cases had used the cubital fossa, seven had also used site(s) on the legs, one had used the foot and one had used the neck. Ten (21%) people had used three areas prior to using the groin. Again in all cases the cubital fossa had been used, nine of the ten had used sites on the legs, six had used the feet and five had used the neck. Five (11%) people reported injecting in four areas prior to the groin. All had used the same four areas, which were the cubital fossa, legs, feet and neck. Thirteen (28%) people had used more than four areas prior to the groin and classed themselves as having used 'everywhere'.

### Why did you start injecting in your groin?

The main reason given for starting to inject in the groin was that 'no other sites were left'. However as many people had not tried all other sites, this was probed further. Further discussion found that in the majority of cases, no other *convenient *sites were perceived to be accessible. Many reported that practises such as the rotation of injecting sites, as advocated in many harm reduction publications, were found to be difficult and unreliable. The risk of 'losing' an injection (missing IV access) through poor injecting technique was considered to be too big a risk, presumably because subcutaneous and intramuscular drug absorption does not provide the same euphoria. Use of the non-dominant hand to administer injections was also reported to be difficult and deter rotation of injecting sites between arms, or require third party assistance. The groin site was reported by most to be convenient, provide quick access, with little mess and less pain than smaller more awkward veins. The gradual formation of sinuses in the groin over time was reported to further facilitate continued use of this site.

### Drugs injected into the groin and equipment used

The most common drug injected by the group was heroin, used by 46 (98%) of interviewees. Nineteen people (40%) injected crack cocaine and eight (17%) injected amphetamine. Twenty four people (51%) currently injected one main drug only into the groin, with 23 injecting heroin and one injecting amphetamine. Twenty people (43%) injected two main drugs into the groin, for 16 of these people their main drugs were heroin and crack cocaine. The remaining four injected heroin and amphetamine. Three people injected three main drugs into the groin and for all these were heroin, crack cocaine and amphetamine. No other drugs were reported to be injected into the groin within the group.

The most common injecting equipment used to access the groin was detachable 1 ml syringes with orange needles (0.5 × 25 mm, 25G) used by 33 people (70%), 11 people (23%) used the same syringes with blue needles (0.6 × 30 mm, 23G) and one person (2%) used green needles (0.8 × 40 mm, 21G). Seven people (15%) used 1 ml insulin syringes. Numbers exceed 100% as four people regularly used more than one type of equipment for groin access.

### Condition of the groin site and history of access problems

Participants were asked whether they were currently or had in the past experienced any problems gaining IV access using the groin site. They were also asked to self-assess the current condition of their groin based on a five point Likert scale: *'very poor', 'poor', 'OK', 'good' or 'very good'*.

Approximately one third of people reported never having had a problem gaining IV access at their groin site (n = 16, 34%). This group comprised 11 males and five females. Five described the current state of their groin site as '*ok*', five said it was '*good*' and six said it was '*very good'*. Their mean length of use of the groin site was 1.1 yrs (SD = 1.2). Approximately two thirds of people had experienced problems with IV access at the groin site on one or more occasions in the past, or were currently experiencing problems (n = 31, 66%). This group comprised 20 males and 11 females. When asked to describe the current condition of their groin two said it was '*very poor'*, seven said it was '*poor*', 14 described it as '*ok*', three said it was '*good*' and five said it was '*very good'*. Their mean length of use of the groin site was 3.3 yrs (SD = 3.8).

When asked to describe the types of access and health problems experienced, a common problem reported was hardened scar tissue occluding the site. This was said to be difficult to penetrate and a cause of needles bending and breaking, causing some people to select longer, thicker needles. Another common problem was swellings in the groin area, accompanied at times by pain. Some people reported infections at the injecting site. Some reported having experienced 'blood clots' or 'DVT' (deep vein thrombosis). It is unknown whether these had been medically diagnosed or treated.

## Discussion

It is of interest that in the overall sample (n = 92) all those who had tried groin injecting (n = 47) continued to do so, despite two thirds having experienced problems with access and a range of health problems at the site. These included reports of infections, hardened tissue, swelling and DVT, which is consistent with problems described in the literature. Further exploration of factors that discourage use of the groin amongst non groin injectors would be of interest. Comparisons between vein health and injecting practices of groin injectors and those who do not inject in the groin would establish protective factors. A longitudinal study is necessary for this to establish the factors that protect and damage the patency of veins. Modifiable protective factors could inform safer injecting advice. The average length of time of groin injecting amongst the sample was 2.6 years with the longest time being 15 years, illustrating that this site of access can be used for considerable time. The formation of sinuses around hardened scar tissue was seen to be an advantage and facilitated continued use, despite posing the risk of breaking needles. Further work to explore responses to problems with the groin site and long-term consequences would be of interest.

Darke et al [7] reported an average time of 10 years from first injection to use of the groin site in their sample of IDUs in Sydney. In this study the average length of time from first injection to use of the groin site was 7 yrs, with more than half (53%) of participants having begun groin injecting within 5 yrs. One theory for the earlier use of the groin site in Bristol is that the use of acidifiers such as citric acid, necessary to dissolve the brown base heroin common in Western Europe, shortens the usable life of veins. Acidifiers are not used in Australia as street heroin is in a soluble form. However further work would be needed to confirm is this theory is true.

A pattern of use of various sites prior to the groin site was found in the majority in this study, similar to the findings of Darke et al [7]. However in this study there were some for whom rapid progression to use of the groin occurred, for example nine participants had only used one site, the cubital fossa, prior to the groin and a further nine had only used two sites. The qualitative data gathered from the sample illustrated that the groin was viewed as 'easy-to-use' with more security of delivering the injection intravenously than other, more awkward sites. This study showed that the groin site was favoured over others for utility and convenience. Other useable sites did potentially exist in many, such as the cubital fossa of the dominant arm, but were viewed as difficult to use and risked loss of the injection. A need for safer injecting information was highlighted in many in this study and practice within the agency has been developed to address this. Future work should examine decision making around use of the groin and whether information on health risks coupled with support to access more awkward peripheral veins can deter use of the groin. However caution is needed not to promote use of sites that require third party assistance, as this may reduce the level of control the IDU has over the injecting process and increase the risks of transmission of hepatitis C and other blood-borne pathogens.

Several points can be learned from this work to inform the delivery of harm reduction messages to this client group:

1. Recognition of the importance of *utility and convenience *when selecting an injecting site. Had this study not enquired about previous sites used and probed those who said they had 'no other sites' left, it may have been wrongly assumed that they did indeed have no useable sites. The identification of the 'utility and convenience' factor and the difficulties in using the non dominant hand for drug injecting has prompted the authors to consider the implementation of structured safer injecting training for IDUs, in order to deter use of the potentially high risk groin site. Such training, run by experienced nurses or anaesthetists, could develop injecting skills amongst IDUs in order to improve injecting techniques and promote the use of other available peripheral sites on the upper limbs. Such services could be integrated within safer injecting facilities.

2. The data on choice of injecting equipment is encouraging, as the majority of participants used detachable needles, which are intended for intravenous access. Most also chose to use short needles (orange), which it is believed to reduce the extent of vascular assault when injecting. This practice has been promoted by needle exchange workers locally. However the identified use of insulin syringes and longer needles (e.g. blue) in some is of concern. Insulin syringes are fragile and intended for subcutaneous use only hence carry a risk of breaking, especially if scar tissue forms that is tough to penetrate. Longer needles may increase the assault on the vascular system or increase the risk of injuring the surrounding nerves or arteries.

3. The contribution of the quantities, frequency of injecting and poly drug use to vascular damage should be studied. Due to tolerance to the effects of many psychoactive drugs, injectors often use increasing quantities and inject with increasing frequency as their injecting careers lengthen. Some also progress to poly drug use. Just over half of this sample (51%) injected one drug only, and in all but one cases this was heroin with the remaining case being amphetamine. However of the remaining 49% (n = 23) who injected more than one drug, 19 were injecting crack cocaine. Cocaine is a potent local anaesthetic and could potentially increase the risks of using the groin site (and other sites) due to lack of sensation on injecting. Further work is needed to quantify and explore these risks and also to assess the longevity of intravenous access in relation to single and poly drug use and frequency of use.

This study focused on the practices of groin site injectors in Bristol using the BDP needle exchange services, illustrating past and current injecting practices, identifying learning points for safer injecting advice delivery and future research. A convenience sample was used and the sample size was dictated by willingness to participate in the given time frame of the study. The results should not be extrapolated to the rest of the UK or those not in contact with BDP.

## Conclusion

This study found, amongst a convenience sample of IDUs in Bristol, that the average time from first injection to use of the groin site was 7 yrs, with the majority having begun use of this site within five years. Reasons for use of the groin site centred on utility, convenience, ease of use and reduced risk of losing the euphoric effects due to extravenous delivery. Several key points were generated to inform the BDP harm reduction service, including the idea of developing safer injecting workshops for IDUs and encouragement that messages on use of equipment were successful. Several areas for future research have been prompted by this study. Groin site injecting is a risky practice that appears to have had little mention in the literature other than the reporting of case studies. The health risks are significant therefore further work to better understand this practice amongst IDUs and how to deter it would be of benefit.

## Competing interests

The author(s) declare that they have no competing interests.

## Authors' contributions

The questionnaire was developed on the basis of discussion between the two authors and staff at BDP. JM, who is employed by BDP, conducted the interviews. JS entered and analysed all the data and drafted the first version of this paper. Both authors contributed towards the revision of this paper and production of the final draft.
